# Supplementary material for: Inhibition of Aldose Reductase by Ginsenoside Derivatives via a Specific Structure Activity Relationship with Kinetics Mechanism and Molecular Docking Study
Source: Molecules. 2022 Mar 25;27(7):2134. doi: 10.3390/molecules27072134 (PMC9000482; doi:10.3390/molecules27072134)
Supplement: Supplementary file 1 [file molecules-27-02134-s001.zip › molecules-1645003-supplementary.pdf]

molecules

MDPI

Supplementary Materials

Inhibition of Aldose Reductase by Ginsenoside Derivatives via a Specific Structure Activity Relationship with Kinetics Mechanism and Molecular Docking Study

Md Yousof Ali [1], Sumera Zaib [2], Susoma Jannat [3], Imtiaz Khan [4], M. Mizanur Rahman [5], Seong Kyu Park [6] and Mun Seog Chang [6,7,*]

[1] Department of Physiology and Pharmacology, Hotchkiss Brain Institute and Alberta Children's Hospital Research Institute, Cumming School of Medicine, University of Calgary, Calgary, AB T2N 4N1, Canada; mdyousof.ali@ucalgary.ca
[2] Department of Biochemistry, Faculty of Life Sciences, University of Central Punjab, Lahore 54590, Pakistan; sumera.biochem@gmail.com
[3] Department of Biochemistry and Molecular Biology, University of Calgary, Calgary, AB T2N 1N4, Canada; jannatacct@gmail.com
[4] Department of Chemistry, Manchester Institute of Biotechnology, The University of Manchester, 131 Princess Street, Manchester M1 7DN, UK; imtiaz.khan@manchester.ac.uk; kimtiaz@hotmail.co.uk
[5] Department of Biotechnology and Genetic Engineering, Faculty of Biological Science, Islamic University, Kushtia 7003, Bangladesh; mmrahmanbtg79@hotmail.com
[6] Department of Prescriptionology, College of Korean Medicine, Kyung Hee University, 26, Kyunghee dae-ro, Dongdaemun-gu, Seoul 02447, Korea; comskp@khu.ac.kr
[7] Qgenetics, Seoul Bio cooperation center 504, 23 Kyunghee dae-ro, Dongdaemun-gu, Seoul 02447, Korea
* Correspondence: mschang@khu.ac.kr; Tel.: +82-2-961-9443

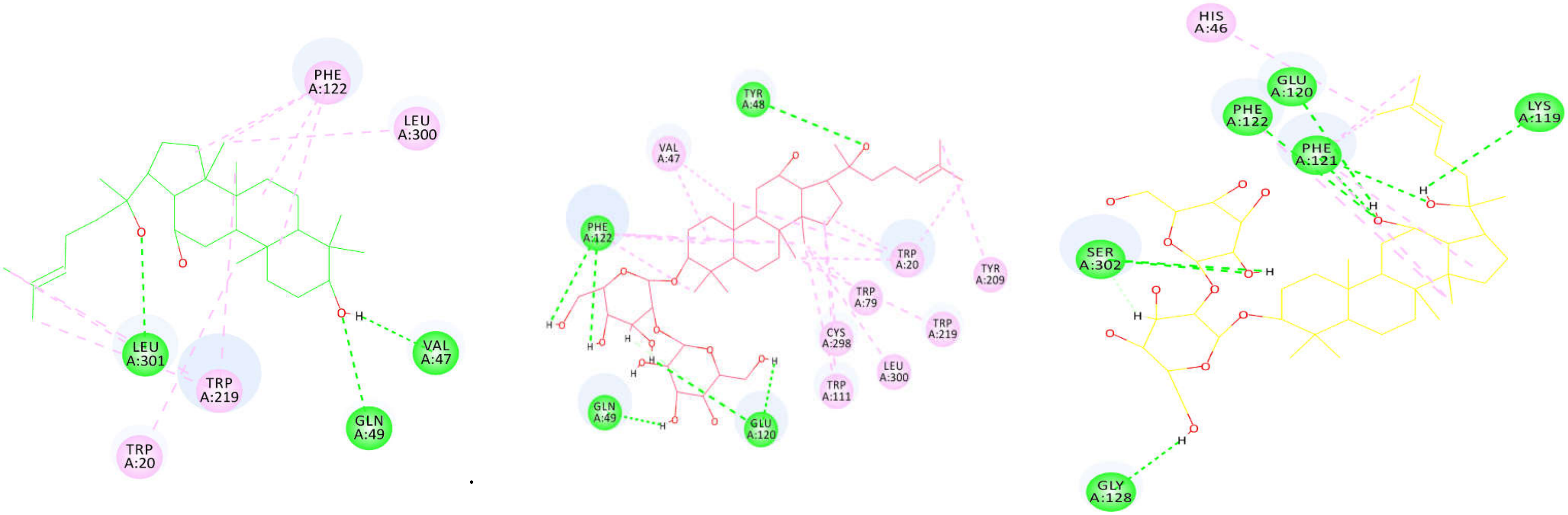
PHE A:122
LEU A:300
LEU A:301
TRP A:219
TRP A:20
GLN A:49
VAL A:47
TYR A:48
VAL A:47
PHE A:122
TRP A:20
TYR A:209
TRP A:79
TRP A:219
CYS A:298
LEU A:300
TRP A:111
GLN A:49
GLU A:120
HIS A:46
GLU A:120
PHE A:122
PHE A:121
LYS A:119
SER A:302
GLY A:128

(**a**) (**b**) (**c**)

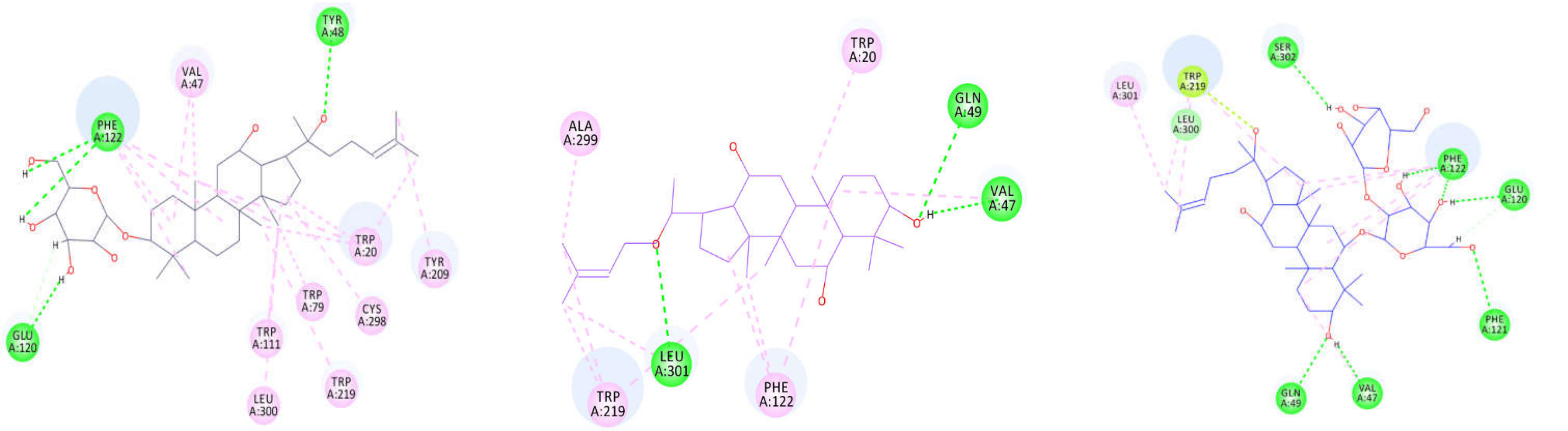
TYR A:48
VAL A:47
PHE A:122
GLU A:120
TRP A:20
TYR A:209
TRP A:79
CYS A:298
TRP A:111
LEU A:300
TRP A:219
TRP A:20
GLN A:49
ALA A:299
VAL A:47
LEU A:301
TRP A:219
PHE A:122
SER A:302
LEU A:301
TRP A:219
LEU A:300
PHE A:122
GLU A:120
PHE A:121
GLN A:49
VAL A:47

(**d**) (**e**) (**f**)

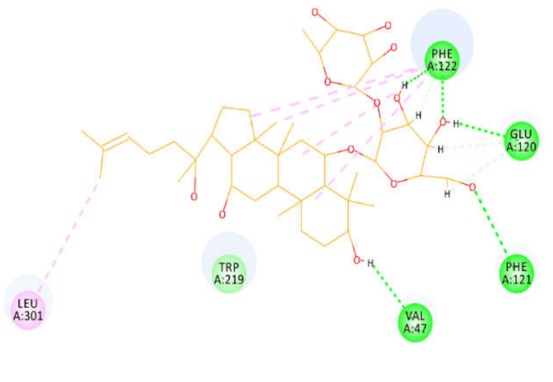

(g)

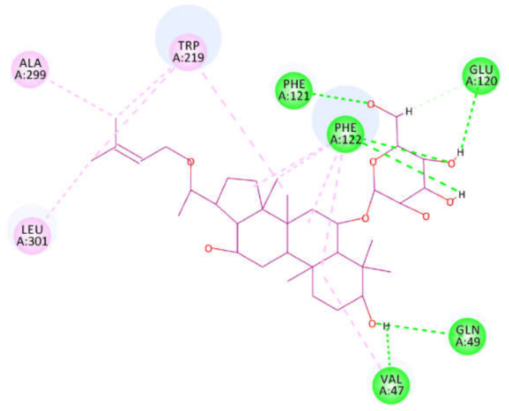

(h)

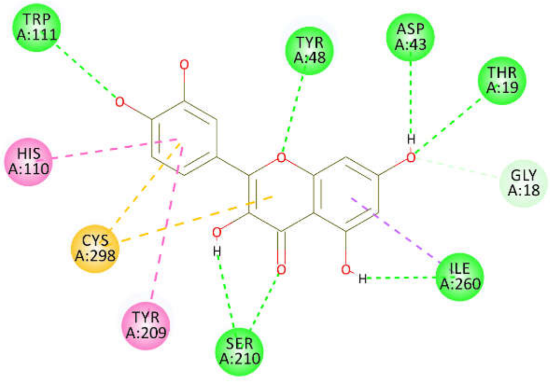

(i)

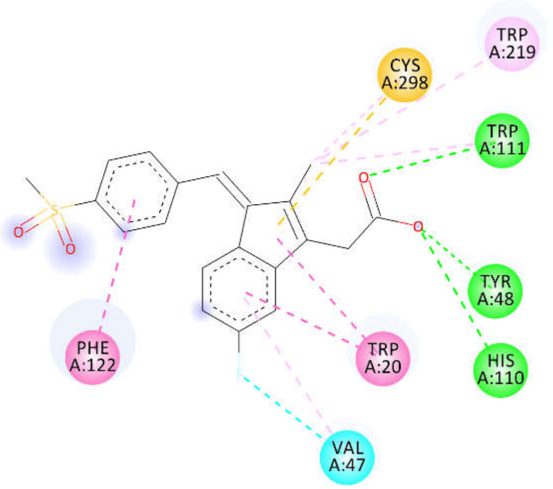

(j)

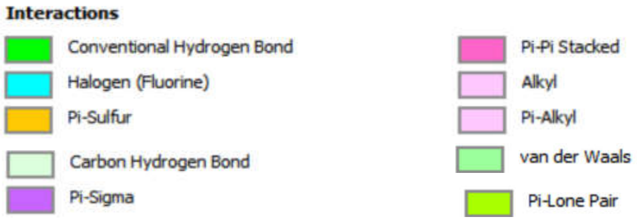

Figure S1. 2D interactions of ginsenosides inside the active pocket of rat lens aldose reductase (RLAR). The interactions are represented by green (conventional hydrogen bonding), yellow (π-sulfur interactions), tea pink (π-π T-shaped and π-π stacked interactions), cyan (fluorine) and yellow (π-sulfur). Protopanaxadiol (**a**), (20*S*) ginsenoside Rg3 (**b**), (20*R*) ginsenoside Rg3 (**c**), ginsenoside Rh2 (**d**), protopanaxatriol (**e**), ginsenoside Rf (**f**), (20*S*) ginsenoside Rg2 (**g**), and ginsenoside Rh1(**h**), quercetin (**i**), and sulindac (**j**).

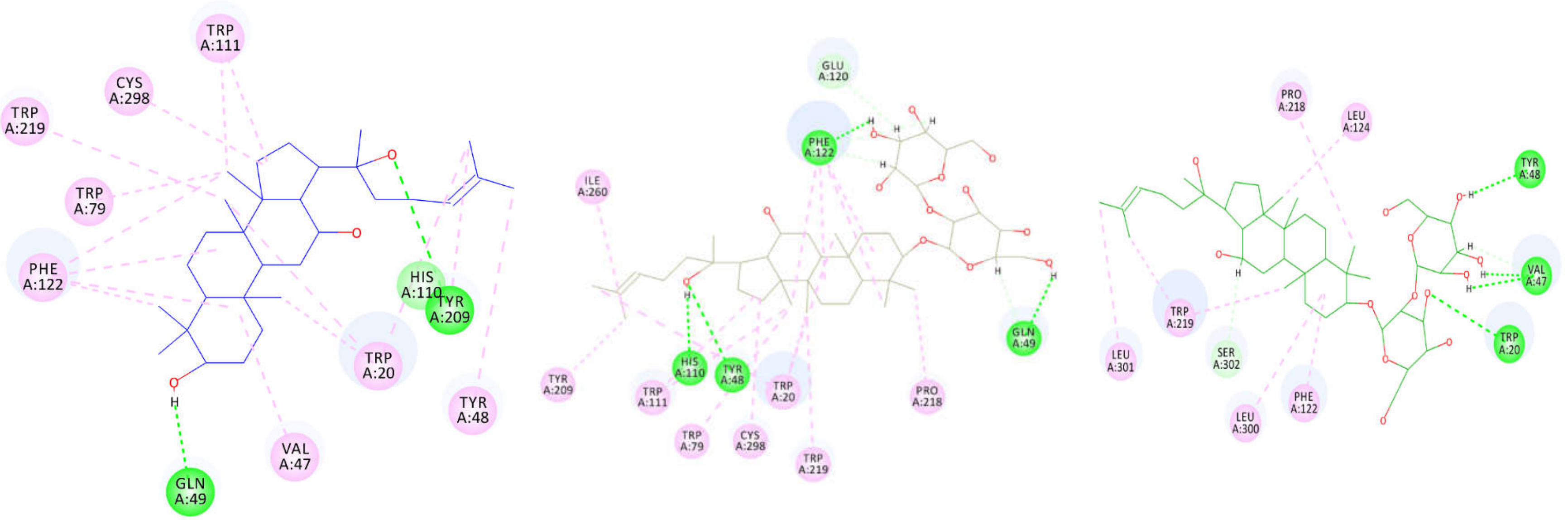
TRP A:111
CYS A:298
TRP A:219
TRP A:79
PHE A:122
HIS A:110
TYR A:209
TRP A:20
TYR A:48
VAL A:47
GLN A:49
GLU A:120
PHE A:122
ILE A:260
HIS A:110
TYR A:48
TRP A:20
TYR A:209
TRP A:111
TRP A:79
CYS A:298
TRP A:219
PRO A:218
GLN A:49
PRO A:218
LEU A:124
TYR A:48
VAL A:47
TRP A:20
TRP A:219
SER A:302
LEU A:301
LEU A:300
PHE A:122

(**a**) (**b**) (**c**)

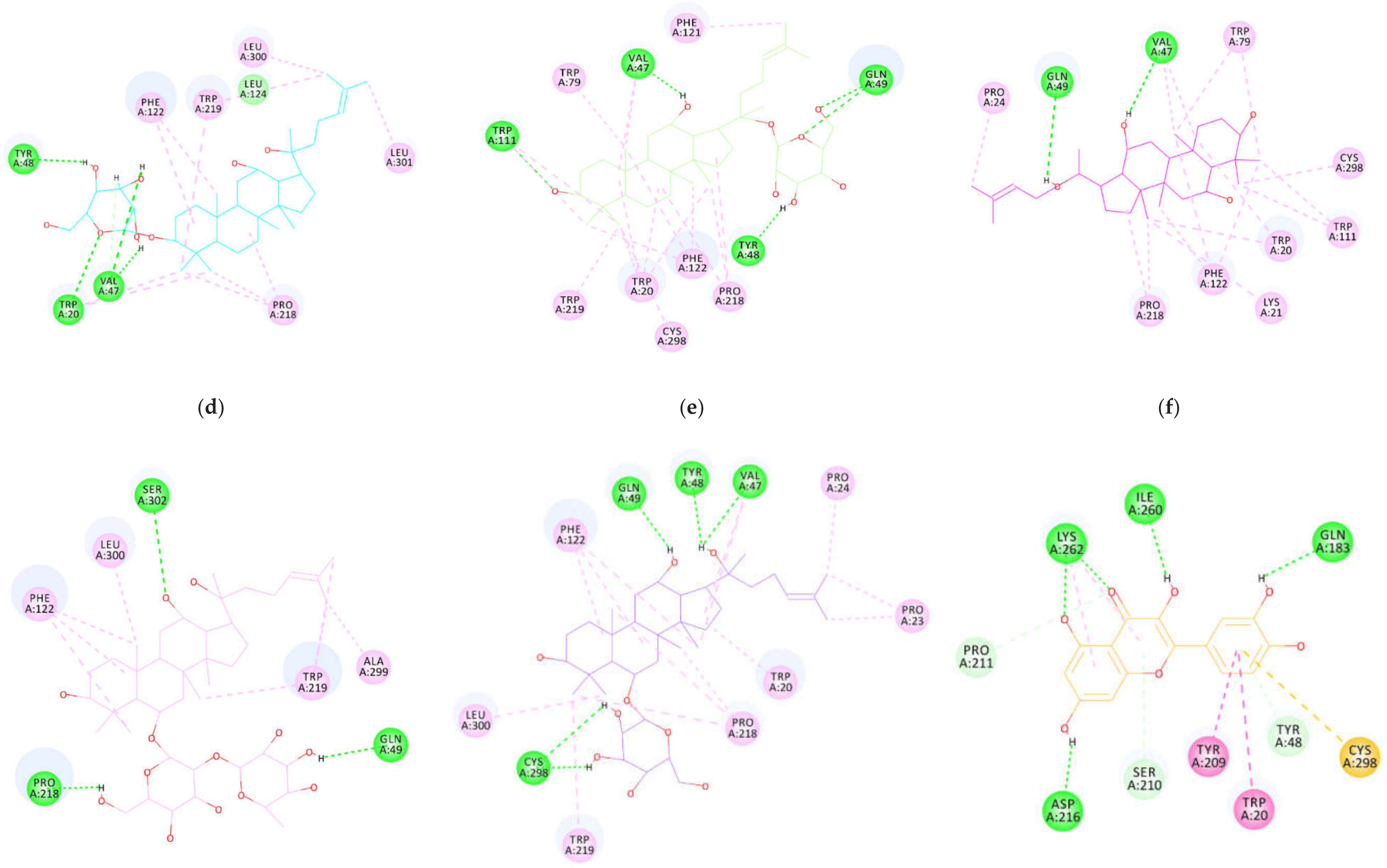
LEU A:300
LEU A:124
PHE A:122
TRP A:219
TYR A:48
LEU A:301
VAL A:47
TRP A:20
PRO A:218
PHE A:121
TRP A:79
VAL A:47
GLN A:49
TRP A:111
TYR A:48
PHE A:122
TRP A:20
PRO A:218
TRP A:219
CYS A:298
TRP A:79
VAL A:47
GLN A:49
PRO A:24
CYS A:298
TRP A:111
TRP A:20
PHE A:122
PRO A:218
LYS A:21
SER A:302
LEU A:300
PHE A:122
TRP A:219
ALA A:299
GLN A:49
PRO A:218
TYR A:48
VAL A:47
GLN A:49
PRO A:24
PHE A:122
PRO A:23
TRP A:20
LEU A:300
PRO A:218
CYS A:298
TRP A:219
ILE A:260
LYS A:262
GLN A:183
PRO A:211
TYR A:48
TYR A:209
CYS A:298
SER A:210
TRP A:20
ASP A:216

(**d**) (**e**) (**f**)

(**g**) (**h**) (**i**)

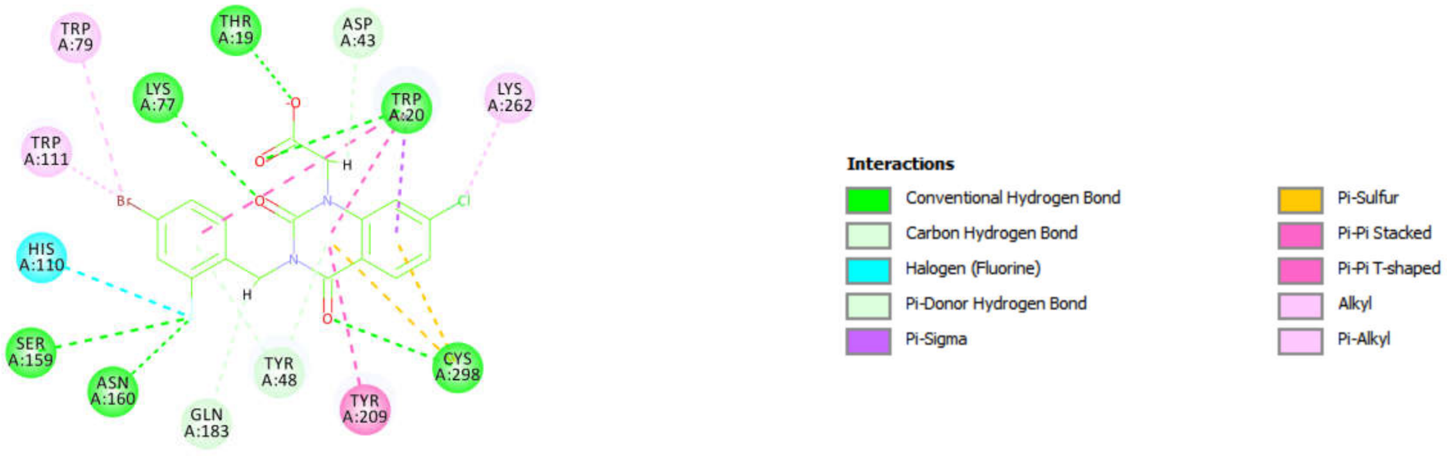

(j)

Figure S2. 2D interactions of ginsenosides inside the active pocket of human aldose reductase (HRAR). The interactions are represented by green (conventional hydrogen bonding), yellow (π-sulfur interactions), tea pink (π-π T shaped and π-π stacked interactions), cyan (fluorine) and yellow (π-sulfur). Protopanaxadiol (a), (20*S*) ginsenoside Rg3 (b), (20*R*) ginsenoside Rg3 (c), ginsenoside Rh2 (d), compound K (e), protopanaxatriol (f), (20*R*) ginsenoside Rg2 (g), ginsenoside Rh1(h), quercetin (i), and zenaresta (j)
